# Boosting the Performance of Electrocatalytic NO Reduction to NH_3_ by Decorating WS_2_ with Single Transition Metal Atoms: A DFT Study

**DOI:** 10.3390/ma18102341

**Published:** 2025-05-17

**Authors:** Mamutjan Tursun, Ayxamgul Abduryim, Chao Wu

**Affiliations:** 1 Xinjiang Key Laboratory of Novel Functional Materials Chemistry, College of Chemistry and Environmental Sciences, Kashi University, Kashi 844000, China; 18709981047@163.com; 2 Frontier Institute of Science and Technology, Xi’an Jiaotong University, Xi’an 710054, China

**Keywords:** nitric oxide electrocatalytic reduction, NH_3_ synthesis, WS_2_ monolayer, density functional theory

## Abstract

Ammonia (NH_3_) is a crucial feedstock in chemical manufacturing. The electrocatalytic NO reduction reaction (eNORR) to NH_3_ represents a promising alternative method for the green production of NH_3_ and for environmental management. This study presents a comprehensive investigation of eNORR properties of single transition metal atoms deposited on WS_2_ nanosheets (TM@WS_2_). Our results indicate that 19 single TM atoms exhibit strong thermal stability. Among these, five specific TM@WS_2_ catalysts—Ti, Mn, Co, Zr, and Hf—demonstrate remarkable eNORR activity, with limiting potentials of 0, −0.19, −0.26, 0, and −0.15 V, respectively. These catalysts effectively suppress the formation of byproducts (N_2_O/N_2_) and the hydrogen evolution reaction (HER), thereby ensuring high NH_3_ selectivity. Our theoretical study confirms that TM@WS_2_ catalysts are highly promising for achieving high activity, selectivity, and stability in eNORR, providing valuable insights for future experimental investigations into efficient NH_3_ production.

## 1. Introduction

Ammonia (NH_3_) serves as a crucial chemical compound not only for the production of fertilizers, pharmaceuticals, and dyes, but also as a significant carrier of carbon-free energy [1,2,3]. Moreover, given its high hydrogen content (17.6 wt%) and its ability to easily condense into a liquid state, NH_3_ is considered an ideal medium for hydrogen energy storage [1,4]. As a result, the synthesis of NH_3_ is of great importance and has garnered widespread attention. Traditionally, the large-scale production of ammonia relies on the Haber–Bosch process, which necessitates extremely high temperatures (400–500 °C) and significant pressures (200–250 bar). However, this widely used process has significant drawbacks, as it depends on fossil fuel combustion and results in the emission of the greenhouse gas CO_2_ (accounting for approximately 3% of global CO_2_ emissions annually) [2,5]. The method of electrocatalytic ammonia synthesis has garnered widespread attention and thorough research as a viable alternative [2,6]. This method operates at room temperature and can utilize electric energy derived from solar or wind power [6]. Therefore, electrocatalytic NH_3_ synthesis is viewed as an environmentally friendly and flexible technique, with nitrogen (N_2_) or nitrogen oxides, such as nitrate (NO_3_^−^) and nitric oxide (NO) serving as its feedstocks [6,7,8,9,10,11,12,13,14,15,16,17].

Despite over a decade of research, the electrocatalytic nitrogen reduction reaction (eNRR) has not yet reached practical implementation, mainly attributed to its insufficient NH_3_ yield and poor Faradaic efficiency. The challenges arise from the robust nature of the N≡N triple bond, which has a bond energy of 914 kJ/mol and makes nitrogen molecules highly unreactive. Additionally, the eNRR faces issues with poor selectivity, as it often competes unfavorably with the hydrogen evolution reaction (HER) [12]. Consequently, there remains an urgent need and significant opportunity to develop exceptionally efficient and specific electrocatalysts to facilitate efficient NH_3_ synthesis.

In addition, flue gases from thermal power plants and industrial boilers typically contain high levels of NO*_x_*, with NO accounting for approximately 95% of these emissions. The significant presence of NO in flue gases poses a serious threat to both the environment and human health [18,19]. Therefore, electrocatalytic NO reduction (eNORR) offers a dual benefit by not only producing valuable NH_3_ but also effectively cleaning the flue gases.

To date, numerous types of catalysts have been investigated for the eNORR [20,21,22,23,24,25,26,27,28,29,30,31,32,33,34]. Noble metal catalysts, in particular, have garnered significant interest due to their intrinsic properties. However, their application is often limited by insufficient activity or prohibitive cost [21,26,27]. In addition to noble metals, transition metal-based composites have also been investigated as potential catalysts for eNORR [20,23,24,25]. Despite these efforts, the durability and selectivity of these catalysts have not yet reached the desired standards. At present, the primary obstacle in the realm of eNORR is to develop catalysts that simultaneously exhibit high activity, excellent durability, and cost-effectiveness.

Single-atom catalysts (SACs) have emerged as promising candidates for the eNORR due to their exceptional atom utilization efficiency and unique geometric and electronic properties [32,33,34]. To date, many substrates are used as single-atom catalysts in eNORR, such as graphene [28,29,30,31,33], transition metal dichalgonides (TMDs) [32,35,36,37], metal mxides [38], carbides (including metal and non-metal carbides) [39,40,41], nitrides (including metal and non-metal nitrides) [42,43,44,45,46], and others [47,48,49,50,51]. However, the inherently high surface energy of single atoms on these substrates tends to drive metal atoms to aggregate into clusters, which, in turn, impedes the formation of highly dispersed single atoms [36,37,38]. To overcome this challenge, it is crucial to pinpoint the optimal substrates that can firmly immobilize and stabilize the single atoms. Recent research has demonstrated that vacancies in support materials can act as efficient binding sites for individual metal atoms, effectively inhibiting their nucleation and aggregation [22,38,39,40].

WS_2_ is an ideal substrate, owing to it is particularly prone to the formation of sulfur vacancies, which provide effective sites for the deposition of single metal atoms [52,53,54]. Experimental studies have shown that loading Fe, Co, Ni, and Cu atoms on WS_2_ at concentrations up to 10% does not induce nucleation [53]. Inspired by these findings, we have designed a WS_2_ nanosheet with a single sulfur vacancy modified by transition metal (TM) atoms, which can serve as highly efficient single-atom catalysts (SACs) for the eNORR.

In this study, we employed DFT calculations to evaluate the electrocatalytic nitric oxide reduction reaction (eNORR) performance of 27 TM single-atom catalysts deposited on WS_2_ (denoted as TM@WS_2_, where TM includes atoms from the 3 d to 5 d periods). First, we assessed the thermal stability of these catalysts by calculating their binding energies. Subsequently, we evaluated their practical viability by comparing the binding energies of synthesized TM@WS_2_ catalysts. After identifying the stable and viable candidates, we examined their ability to adsorb and activate NO. We further explored the eNORR mechanisms leading to NH_3_ production and evaluated their catalytic performance in competing reactions, such as the formation of N_2_O or N_2_ and the hydrogen evolution reaction (HER). Finally, we elucidated the origin of activity for the promising eNORR candidates through detailed electronic structure analysis.

## 2. Materials and Methods

We conducted spin-polarized periodic density functional theory (DFT) calculations using the Vienna ab initio simulation package (VASP version 5.4.4) [55]. The electron exchange and correlation effects were described using the generalized gradient approximation (GGA) with the Perdew–Burke–Ernzerhof (PBE) functional [56]. The projector augmented wave (PAW) method was applied, and a plane-wave cutoff energy of 450 eV was set [57]. To incorporate van der Waals interactions between adsorbates and substrates, the DFT-D3 correction was employed [58]. A 4 × 4 × 1 supercell was created, and a 15 Å vacuum layer was introduced to reduce interactions between WS_2_ nanosheets and their periodic images. The k-point grid of 4 × 4 × 1 was used for Brillouin zone sampling [58]. All atoms were allowed to relax freely without any constraints, and the calculations were converged to an energy tolerance of 10^−4^ eV and a residual force tolerance of 0.02 eV Å^−1^. The binding energies (E_b_) of TM on WS_2_ were calculated using the following formula [59,60]:

(1) $$E_{b}=E_{\mathrm{TM}@{\mathrm{WS}}_{2}}-E_{\mathrm{defective}\text{-}{\mathrm{WS}}_{2}}+\mu_{\mathrm{TM}}$$

Here, E_TM@WS2_ signifies the total energy of the transition metal (TM) atoms deposed on WS_2_, while E_defective-WS2_ refers to the total energy of WS_2_ with a single sulfur vacancy. μ_TM_ denotes the chemical potential of the TM atoms. The Gibbs free energy (ΔG) for each step of eNORR was determined using the following equation [33,34]:

ΔG = ΔE + ΔE_ZPE_ − TΔS + ΔG_pH_ (2)

In this equation, ΔE denotes the energy difference between the two intermediates involved in each elementary reaction step. This value can be directly obtained from the DFT results. ΔE_ZPE_ and ΔS represent the changes in zero-point energy and entropy, respectively, at room temperature (T = 298.15 K). These values were calculated using vibrational frequencies through the VASPKIT program [61]. The ΔG correction for pH (ΔG_pH_) was determined by:

ΔG_pH_ = k_B_T × pH × ln10 (3)

In this case, the pH was fixed at 0. The influence of the solvent (water) on the ΔG was assessed via implicit solvent model, which is integrated within the VASP computational framework [62].

The limiting potential (U_L_) acts as an activity indicator, representing the minimum electrical potential required to make the ΔG of all elementary steps in the reaction exothermic (i.e., to ensure that ΔG decreases for each step). It is calculated using the following equation [33,34]:

U_L_ = −max (∆G_1_, ∆G_2_, ∆G_3_, ∆G_4_…, ∆G_i_)/e (4)

In this equation, ΔG_i_ is the free energy variation corresponding to each specific intermediate step within the overall eNORR process.

## 3. Results

### 3.1. Stability of TM@WS_2_

**Figure 1:**
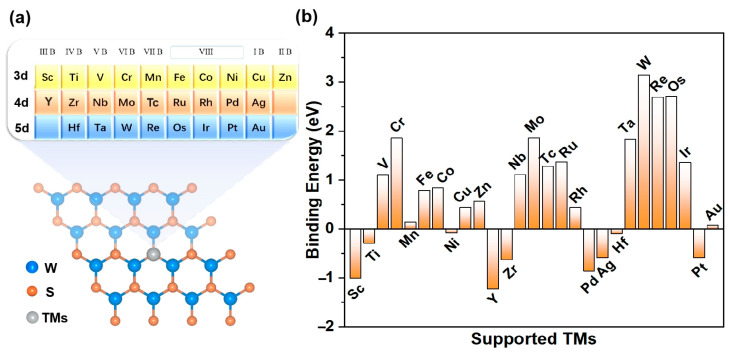
(**a**) The modeled structure of TM@WS_2_ catalysts; (**b**) E_b_ values of TM@WS_2_ catalysts.

The specific TM@WS_2_ model under investigation is depicted in Figure 1a. In order to assess the stability and experimental viability of the TM@WS_2_ catalysts, we first calculated their binding energies (E_b_). The E_b_ values of several synthesized TM@WS_2_ catalysts (where TM represents Fe, Co, and Cu) were used as reference values to estimate the stability of other catalysts [53]. Given these values, an E_b_ of 0.84 eV can be used as a preliminary benchmark to evaluate the stability of other unsynthesized TM@WS_2_ catalysts. Figure 1 also shows that, among the 27 TM@WS_2_ catalysts examined, 11 metal atoms —specifically, V, Cr, Nb, Mo, Tc, Ru, Ta, W, Re, Os, and Ir—exhibit E_b_ values greater than 0.84 eV, suggesting that they are considered unstable; these have been excluded from further analysis. Conversely, the remaining 16 TM atoms, which possess E_b_ values below 0.84 eV, are deemed more probable candidates for stable TM@WS_2_ catalysts.

In order to conduct a more comprehensive evaluation of the thermodynamic stability of the 16 transition metal (TM) atoms on the WS_2_ surface, we chose the Zn@WS_2_ catalyst as a representative case for performing ab initio molecular dynamics (AIMD) simulations. This selection was based on the fact that the binding energy (E_b_) of Zn@WS_2_ is 0.56 eV, which is above 0 eV. The AIMD results are presented in Figure S1. Upon reaching a stable state at 500 K, the Zn@WS_2_ catalyst exhibited no substantial structural distortions or displacements of the Zn atom, thereby confirming its robust thermodynamic stability.

**Figure 2:**
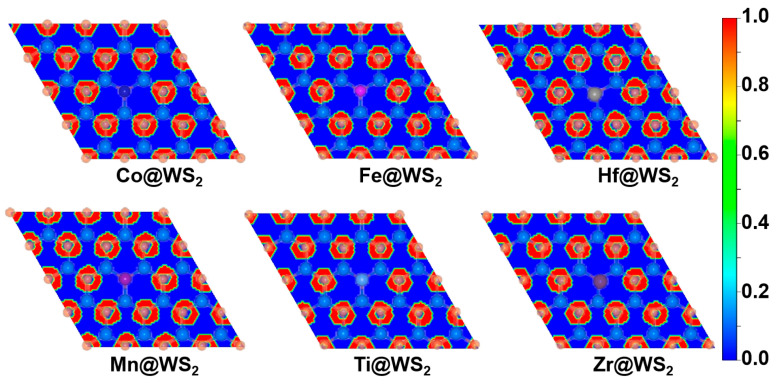
Electron localization function (ELF) plots of TM@WS_2_ catalysts.

By calculating the electron localization function (ELF), we further reveal the bonding characteristics and stability. The closer the ELF value is to 1, the higher the density of covalent bonds and lone pairs of electrons. As shown in Figure 2, there is a high electron distribution between the metal atoms and sulfur atoms (S), indicating that the TM-S bond is a stable covalent bond. Therefore, the TM@WS_2_ catalysts possess good thermodynamic stability.

### 3.2. NO Adsorption

**Figure 3:**
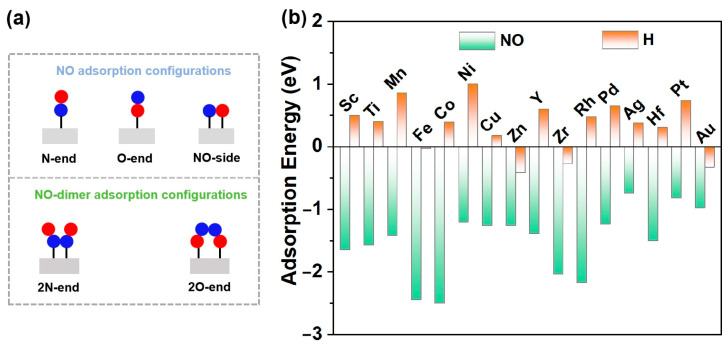
(**a**) Possible NO adsorption configurations both high and low NO concentration; (**b**) NO and H adsorption over 19 TM@WS_2_ catalysts.

NO adsorption and activation are the first steps in eNORR and play a crucial role in the subsequent reaction [32]. There are three different adsorption configurations of the NO molecule: N-end, O-end, and NO-side (as shown in Figure 3). Among the 16 TM@WS_2_ catalysts screened, the N-end adsorption configuration exhibited the most negative adsorption energy compared to the other configurations (Table S1), indicating that it is the most stable configuration. Notably, the NO-side configuration was unstable and spontaneously converted to the N-end configuration on several TM@WS_2_ catalysts, including Co@WS_2_, Ni@WS_2_, Cu@WS_2_, Zn@WS_2_, Rh@WS_2_, Pd@WS_2_, Ag@WS_2_, Pt@WS_2_, and Au@WS_2_. Therefore, our focus is primarily on the N-end configuration.

In an aqueous environment, the hydrogen evolution reaction (HER) emerges as a predominant competing process. In the first step, the reactants (here NO and H) are adsorbed on to the catalysts to initiate the reaction. This competition determines whether eNORR or HER is prioritized. Therefore, following a similar approach, we undertook a comparative analysis of the adsorption energies of of H and NO on 16 catalysts. As shown in Figure 3b, NO adsorption is significantly stronger than H adsorption across all 16 catalysts. This indicates that H adsorption is hindered, thereby favoring eNORR over HER.

### 3.3. eNORR Mechanism and Evaluation of Activity

**Figure 4:**
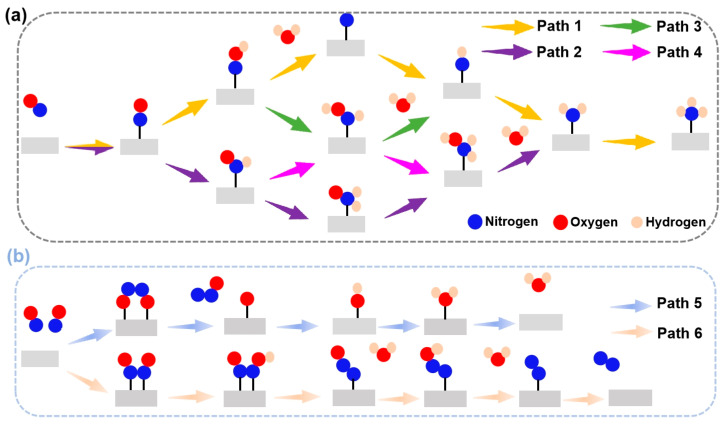
Schematic depiction of the probable reaction pathways for the eNORR. (**a**) Pathways of NO reduction to NH_3_; (**b**) Pathways of NO reduction to N_2_/N_2_O.

The eNORR reaction involves multiple reaction pathways (see Figure 4). The selectivity of the eNORR process is significantly influenced by NO concentration. At low NO concentrations, NH_3_ serves as the final product (as shown in Figure 4a), while at high NO concentrations, N_2_ and N_2_O are formed due to dimerization of NO (N_2_O_2_). In this scenario, since NH_3_ is the target product, a series of distinct pathways are followed and each pathway involves five protonation steps to yield NH_3_. At high NO concentrations, multiple two-pathway branches emerge, with N_2_ and N_2_O being formed through dimerization [26].

**Figure 5:**
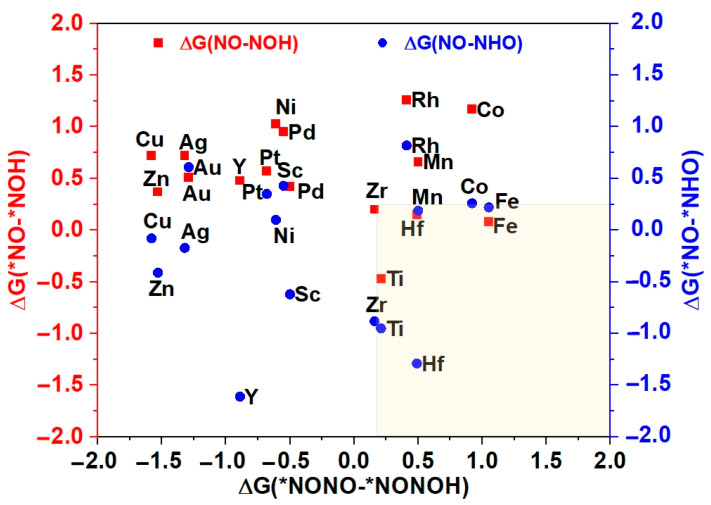
The Gibbs free energy differences of first protonation steps for NH_3_ vs. N_2_. * represents the adsorption states of reaction species.

The potential and efficiency of the eNORR reaction is typically evaluated based on its potential determination step (PDS), which is marked by the most substantial increase in Gibbs free energy change (∆G) along the reaction pathway. In this context, processes such as the initial protonation of *NO to form *NOH or *NHO, and the protonation of *NONO to yield *NONOH, are commonly considered as the PDS [63]. To further evaluate the catalytic activity and product selectivity of the 16 catalysts, the ∆G values for the initial hydrogenation steps were calculated, specifically for the pathways from *NO→*NOH (*NO→*NHO) to NH_3_ and from *NONO→*NONOH to N_2_. As illustrated in Figure 5, for the Ti@WS_2_, Mn@WS_2_, Fe@WS_2_, Co@WS_2_, Zr@WS_2_, and Hf@WS_2_ catalysts, the ∆G values of PDS for the *NO→*NOH or *NO→*NHO steps are lower than those for the *NONO→*NONOH step, indicating that these six catalysts preferentially form NH_3_, making them potential candidate catalysts.

**Figure 6:**
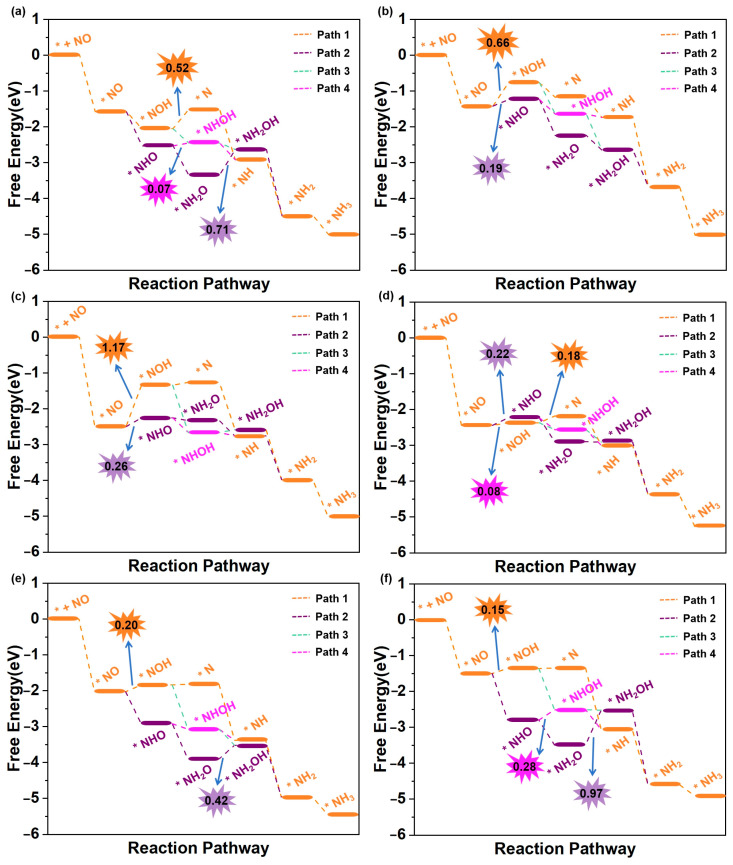
Free energy diagrams of eNORR to NH_3_ over six TM@WS_2_ catalysts. (**a**) Ti@WS_2_; (**b**) Mn@WS_2_; (**c**) Co@WS_2_; (**d**) Fe@WS_2_; (**e**) Zr@WS_2_; (**f**) Hf@WS_2_.

We then subsequently evaluated the eNORR activity of Ti@WS_2_, Mn@WS_2_, Fe@WS_2_, Co@WS_2_, Zr@WS_2_, and Hf@WS_2_ catalysts for NH_3_ synthesis. The eNORR free energy profiles for each reaction pathway of these six catalysts were constructed and are shown in Figure 6a–f. The potential determining step (PDS) for each pathway was identified and labelled, providing a clear indication of the most favorable reaction pathway and associated potential barriers.

The eNORR process is initiated by the adsorption of NO on the catalyst surface, resulting in the formation of the *NO species. Following this, protons from the electrolyte approach and interact with the *NO species, driving the formation of *HNO or *NOH. Subsequently, a series of hydrogenation steps occur, ultimately resulting in the formation of NH_3_. As shown in Figure 6a, the most favorable reaction pathway for the Ti@WS_2_ catalyst is Path3. Along this pathway, all elementary steps are exergonic (∆G < 0 eV), indicating that the eNORR process occurs spontaneously. Consequently, the U_L_ of the Ti@WS_2_ catalyst is 0 V. As shown in Figure 6b,c, for both the Mn@WS_2_ and Co@WS_2_ catalysts, Path2 is selected as the most favorable reaction pathway. In this pathway, the eNORR process can occur after overcoming a potential barrier (corresponding to ∆G of *NO→*NHO) of 0.19 eV for Mn@WS_2_ and 0.26 eV for Co@WS_2_. For Fe@WS_2_ (Figure 6d), Path3 emerges as the most favorable pathway, featuring a minimal energy barrier of 0.08 eV during the transition from *NO to *NHO. For the Zr@WS_2_ catalyst (Figure 6e), the most favorable reaction pathway is Path4, along which all elementary steps are exergonic, suggesting that the eNORR proceeds spontaneously at 0 V electrolyte potential. For the Hf@WS_2_ catalyst, as shown in Figure 6f, the most favorable pathway is identified as Path4, with a barrier height of 0.15 eV. In particular, in an acidic environment, the transformation of NH_3_ into NH_4_^+^ proceeds rapidly, primarily because this process releases a significant amount of energy [22,26,35]. As a result, the desorption of NH_3_ is not taken into account when evaluating the performance of all six catalysts.

In summary, the limiting potentials (U_L_) of the Ti@WS_2_, Mn@WS_2_, Fe@WS_2_, Co@WS_2_, Zr@WS_2_, and Hf@WS_2_ catalysts are 0, −0.19, −0.26, −0.08, 0, and −0.15 V, respectively. These values are either equivalent to or significantly lower than those of reported for other single-atom catalysts. For ease of comparison, we have compiled the limiting potentials from our study and those reported in the literature in Table S2.

In order to further elucidate the effects of TM atoms modification, the eNORR activity of WS_2_ with single sulfur vacancies was investigated. As demonstrated in Figure S2, the most favorable reaction pathway is Path4, which involves a relatively low potential barrier of 0.96 eV for the *NHO→*NHOH step, corresponding to U_L_ of −0.96 V. In comparison, TM depositing on sulfur vacancy site significantly enhances the catalytic activity of WS_2_. Specifically, the U_L_ values for the TM@WS_2_ catalysts exhibit a notable increase from −0.96 V for WS_2_ to 0 V for Ti@WS_2_, −0.19 V for Mn@WS_2_, −0.26 V for Fe@WS_2_, −0.08 V for Co@WS_2_, 0 V for Zr@WS_2_, and −0.15 V for Hf@WS_2_. These observations demonstrate that TM atoms modification substantially improves the eNORR activity of WS_2_.

**Figure 7:**
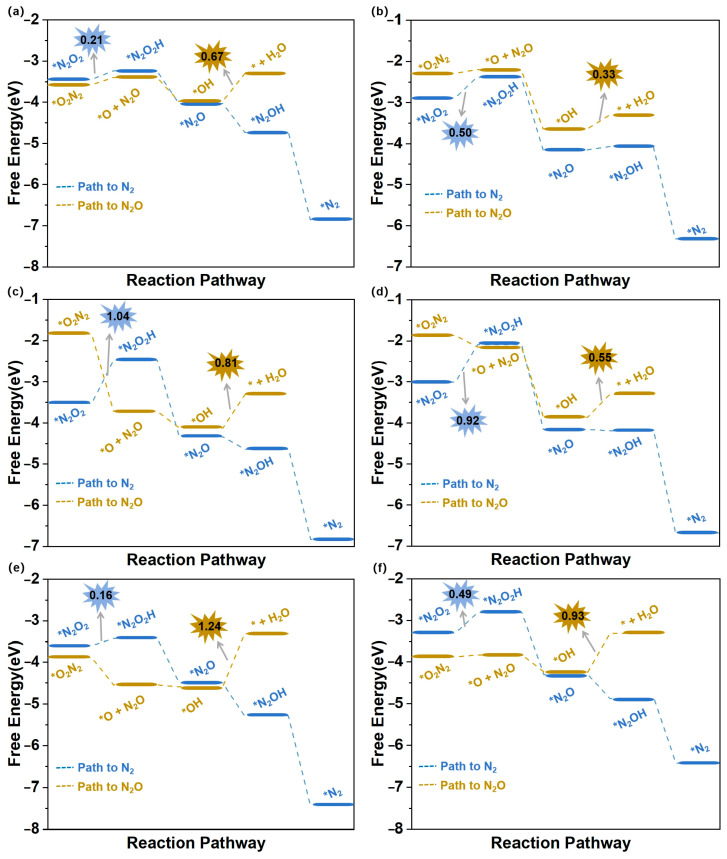
Free energy diagrams of eNORR to N_2_O/N_2_ over six TM@WS_2_ catalysts. (**a**) Ti@WS_2_; (**b**) Mn@WS_2_; (**c**) Fe@WS_2_; (**d**) Co@WS_2_; (**e**) Zr@WS_2_; (**f**) Hf@WS_2_.

During the eNORR process, N_2_O and N_2_ can be produced as by-products due to the formation of the NO dimer (N_2_O_2_), as shown in Figure 3a. Therefore, we investigated the NO-to-N_2_O and NO-to-N_2_ pathways (Path5 and Path6 in Figure 4b) on the above six catalysts from the 2N-end (*N_2_O_2_) and 2O-end (*O_2_N_2_) configurations of N_2_O_2_, respectively. Figure 7a–f illustrate the energy diagrams for the formation of N_2_O and N_2_ on Ti@WS_2_, Mn@WS_2_, Fe@WS_2_, Co@WS_2_, Zr@WS_2_, and Hf@WS_2_ catalysts; during the NO-to-N_2_O process, starting from*O_2_N_2_ configurations, it is evident that all of these six catalysts face potential barriers (in the process of *OH→* + H_2_O step) of 0.67, 0.33, 0.81, 0.55, 1.24, and 0.93 eV, respectively. This indicates that the conversion of *OH to *+H_2_O is a rather challenging step for these catalysts. For the N_2_ formation process, over Ti@WS_2_, Mn@WS_2_, Fe@WS_2_, Co@WS_2_, Zr@WS_2_, and Hf@WS_2_ catalysts, the first hydrogenation of first step, namely the *N_2_O_2_-*N_2_O_2_H step, is the PDS step, and corresponding potential barriers are 0.21, 0.50, 1.04, 0.92, 0.16, and 0.49 eV, respectively. Based on the above results, the energy barriers of N_2_O and N_2_ formation are higher than those of NH_3_ formation. Consequently, although under conditions of high NO concentration, the formation of N_2_O and N_2_ can be effectively suppressed on these six catalysts. Hence, these catalysts demonstrate high selectivity for NH_3_ production.

### 3.4. Selectivity Analysis

**Figure 8:**
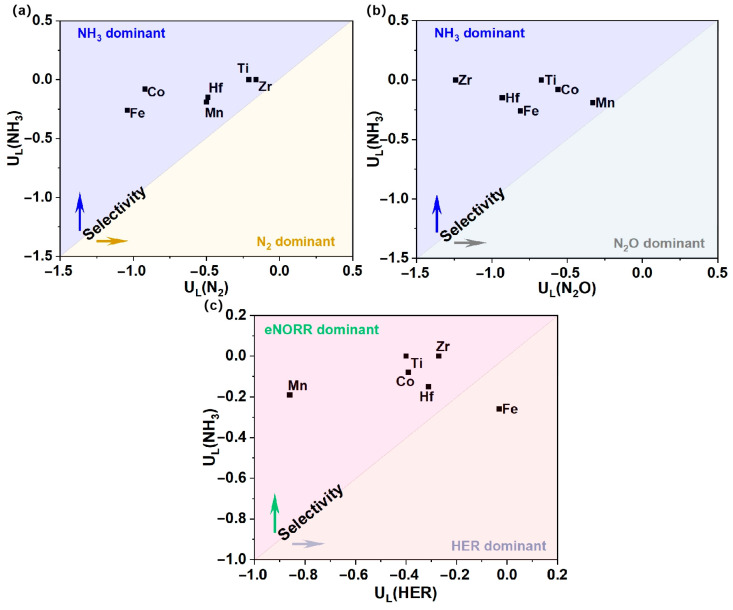
Selectivity for NH_3_ production vs. N_2_O/N_2_ and HER on TM@WS_2_ catalysts. (a) U_L_(NH_3_) vs. U_L_(N_2_); (b) U_L_(NH_3_) vs. U_L_(N_2_O); (c) U_L_(NH_3_) vs. U_L_(HER).

To more effectively elucidate the product selectivity of NH_3_ and N_2_O/N_2_, as well as the HER, for the Ti, Mn, Fe, Co, Zr, and Hf@WS_2_ catalysts, we have constructed a graph depicting the differences in the limiting potentials between NO reduction to NH_3_ and N_2_O/N_2_, as well as the HER. As shown in Figure 8a,b, the Ti, Mn, Fe, Co, Zr, and Hf@WS_2_ catalysts exhibit a pronounced preference for NH_3_ production over N_2_O and N_2_. Meanwhile, as depicted in Figure 8c, with the exception of the Fe@WS_2_ catalyst, the remaining five catalysts—Ti, Mn, Co, Zr, and Hf@WS_2_—maintain a pronounced selectivity for the eNORR over the HER, thereby highlighting their exceptional selectivity for NH_3_ production.

### 3.5. Pourbaix Diagram Analysis

**Figure 9:**
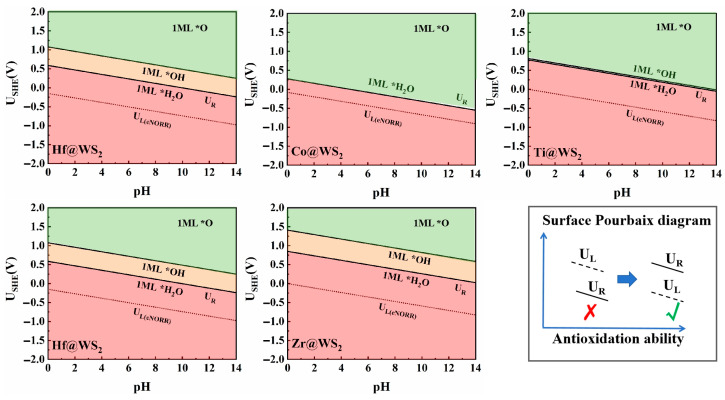
Surface Pourbaix diagrams of TM@WS_2_ catalysts.

Since eNORR typically occurs under solution conditions, hydroxyl groups (*OH), oxygen groups (*O), or water molecules (*H_2_O) present in the solution may adsorb onto the active sites, thereby reducing the activity of catalysts. The Pourbaix diagram, which provides detailed information about the catalyst surface under different pH values and applied voltages [64,65], is a valuable tool for evaluating the electrochemical stability of catalysts. Thus, we employed the Pourbaix diagram to evaluate the electrochemical stability of the TM@WS_2_ catalysts. As shown in the Figure 9, the minimum redox potentials (U_R_) required to eliminate *OH/*O on the surfaces of the Mn@WS_2_, Hf@WS_2_, Zr@WS_2_, Co@WS_2_, and Ti@WS_2_ catalysts are 0.36 V, 0.59 V, 0.85 V, 0.28 V, and 0.77 V, respectively. When the value of U_R_ is greater than U_L_, the electrocatalysts exhibit significant oxidation resistance, preventing the adsorption of *OH/*O on the catalyst surface. This, in turn, facilitates the adsorption of NO molecules on the active sites, thereby promoting the subsequent eNORR process. Notably, for the Mn@WS_2_, Hf@WS_2_, Zr@WS_2_, Co@WS_2_, and Ti@WS_2_ catalysts, the value of U_R_ is greater than the corresponding U_L_. This indicates that these catalysts possess both stability and high selectivity for eNORR. Therefore, they can serve as stable and promising candidates for eNORR applications.

### 3.6. Revealing the Origin of Activity

**Figure 10:**
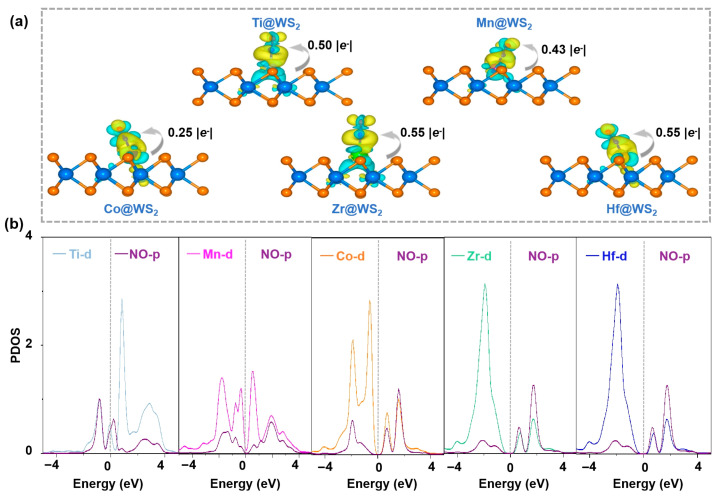
(**a**) The charge density difference maps of five TM@WS_2_ catalysts. In these visualizations, the isosurface threshold is 0.003 e/Å^3^, yellow regions signify areas of charge accumulation, while cyan regions indicate charge depletion; (**b**) partial density of states (PDOS) of NO adsorbed on five TM@WS_2_ catalysts.

The initial stages of the eNORR process are highly contingent upon the adsorption and activation of NO, as these steps serve as the cornerstone for the entire reaction mechanism. To elucidate the origin of eNORR activity, we performed detailed electronic structure analyses to illustrate the activation of *NO. As depicted in Figure 10a, the analysis of electron density difference maps for five TM@WS_2_ catalysts reveals substantial charge redistribution occurring between the adsorbed *NO species and the catalyst substrates, primarily characterized by the accumulation of electrons on the *NO. The Bader charge analysis provides additional validation for this observation, demonstrating that the quantity of electrons transferred from the TM@WS_2_ catalysts to the *NO species varies among different transition metals: 0.50 for Ti, 0.43 for Mn, 0.25 for Co, 0.55 for Zr, and 0.55 for Hf.

A detailed analysis was conducted on the partial density of states (PDOS) for the five catalyst candidates. As shown in Figure 10b, there is a clear overlap between the p orbital of the *NO species and the d orbitals of the Ti, Mn, Co, Zr, and Hf atoms. This overlap indicates a strong interaction between the d orbitals of these metal atoms and the p orbital of *NO, which ultimately promotes the activation (weakening) of the N-O bond in the NO molecule.

The analysis of the crystal orbital Hamiltonian population (COHP) and the integrated crystal orbital Hamiltonian population (ICOHP) further supports the above findings. These metrics have been established as effective indicators for characterizing the level of NO activation [25,60]. The quantitative determination of ICOHP values is achieved by integrating the energy up to the Fermi level. Typically, a more negative ICOHP value signifies a stronger binding interaction.

**Figure 11:**
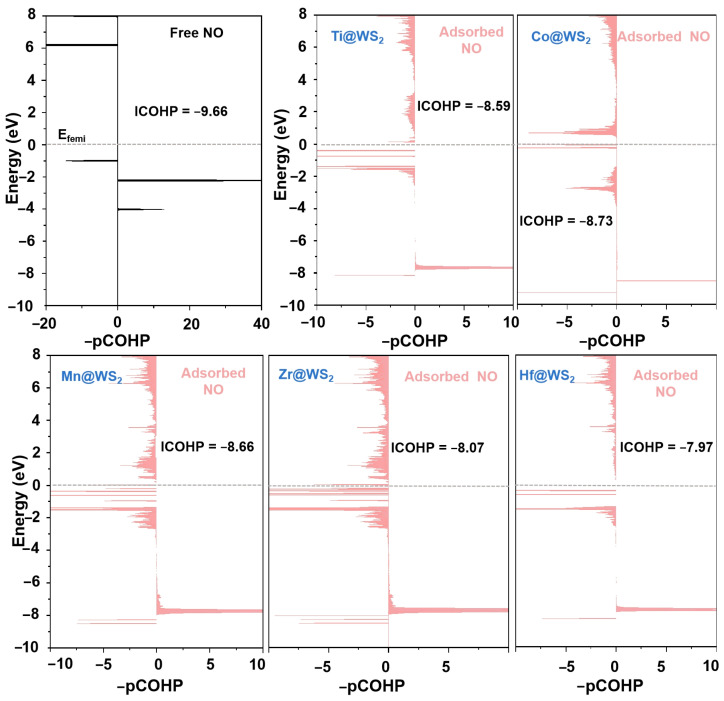
Projected COHP (-pCOHP) analysis of the N-O bond in free NO molecule (dark) versus that in the *NO species (light red) on five TM@WS_2_ catalysts.

As demonstrated in Figure 11, upon NO adsorption, the COHP of the adsorbed *NO exhibits a greater number of antibonding states compared to that of the isolated NO molecule. Additionally, the ICOHP (N-O) values for the N-O bond of *NO adsorbed on Ti, Mn, Co, Zr, and Hf@WS_2_ catalysts are −8.59, −8.66, −8.73, −8.07, and −7.97, respectively. It is evident that this is less pronounced in negativity compared to that of the isolated NO molecule (−9.66), indicating a substantial weakening of the N-O bond upon adsorption.

## 4. Conclusions

In summary, a range of single-atom catalysts comprising 27 transition metal (TM) atoms deposited on single sulfur vacancy sites of WS_2_ nanosheets have been successfully designed and their performance in eNORR evaluated using DFT calculations. The results indicate that 19 TM atoms exhibit potential stability. In terms of eNORR activity for NH_3_ production, the five TM@WS_2_ catalysts—specifically Ti@WS_2_, Mn@WS_2_, Co@WS_2_, Zr@WS_2_, and Hf@WS_2_—show remarkable potential, with their U_L_’s being 0.00, −0.19, −0.26, 0.00, and −0.15 V, respectively. Moreover, by comparing the differences in the limiting potentials for NO reduction to NH_3_ and N_2_O/N_2_, as well as the HER, we reveal their exceptional selectivity for NH_3_ production.

This study is significant for both environmental and energy applications, as it provides a sustainable route for ammonia synthesis and reduces NO emissions through electrocatalytic NO reduction. The identified TM@WS_2_ catalysts, with high activity, selectivity, and stability, are promising for experimental validation. However, limitations exist due to the idealized models and approximations used in DFT calculations, which may not fully reflect real-world complexities such as impurities and solvent conditions. Therefore, close collaboration between theoretical and experimental researchers is essential to validate and refine these predictions.

Overall, this work highlights the potential of TM@WS_2_ catalysts for efficient NO removal and sustainable NH_3_ synthesis, emphasizing the need for further experimental research to realize their practical application.

## Data Availability

The original contributions presented in this study are included in the article/Supplementary Materials. Further inquiries can be directed to the corresponding authors.

## Supplementary Materials

The following supporting information can be downloaded at: https://www.mdpi.com/article/10.3390/ma18102341/s1. Figure S1: Ab initio molecular dynamics (AIMD) simulations at 500 K forZn@WS_2_ catalysts; Figure S2: Free energy diagrams of eNORR over undoped defective WS_2_ catalyst; Table S1: The Gibbs energies of NO adsorptipon on TM@WS_2_ catalysts via different configurations (eV); Table S2: Limiting potentials of eNORR toward NH_3_ over different single atom catalysts. References [28,30,32,34,37,38,40,42,44,45,46,47,48,51,66,67,68] are cited in the Supplementary Materials.

## Abbreviations

The following abbreviations are used in this manuscript:

eNORR	Electrocatalytic nitric oxide reduction
TM	Transition metal
HER	Hydrogen evolution reaction
eNO_3_RR	Electrocatalytic nitrate reduction
eNRR	Electrocatalytic N_2_ reduction reaction
SACs	Single-atom catalysts
E_b_	Binding energies

## References

[1] Christensen C.H., Johannessen T., Sørensen R.Z., Nørskov J.K. (2006). Towards an ammonia-mediated hydrogen economy?. Catal. Today.

[2] Soloveichik G. (2019). Electrochemical synthesis of ammonia as a potential alternative to the Haber–Bosch process. Nat. Catal..

[3] MacFarlane D.R., Cherepanov P.V., Choi J., Suryanto B.H.R., Hodgetts R.Y., Bakker J.M., Ferrero Vallana F.M., Simonov A.N. (2020). A Roadmap to the Ammonia Economy. Joule.

[4] Lin Q.F., Jiang Y.M., Liu C.Z., Chen L.W., Zhang W.J., Ding J., Li J.G. (2021). Instantaneous hydrogen production from ammonia by non-thermal arc plasma combining with catalyst. Energy Rep..

[5] Wang M., Khan M.A., Mohsin I., Wicks J., Ip A.H., Sumon K.Z., Dinh C.-T., Sargent E.H., Gates I.D., Kibria M.G. (2021). Can sustainable ammonia synthesis pathways compete with fossil-fuel based Haber–Bosch processes?. Energy Environ. Sci..

[6] Mohan N.G., Ramanujam K. (2024). Electrocatalysts for ammonia synthesis: How close are we to the Haber-Bosch process?. Curr. Opin. Electrochem..

[7] Liu H.-M., Han S.-H., Zhao Y., Zhu Y.-Y., Tian X.-L., Zeng J.-H., Jiang J.-X., Xia B.Y., Chen Y. (2018). Surfactant-free atomically ultrathin rhodium nanosheet nanoassemblies for efficient nitrogen electroreduction. J. Mater. Chem. A.

[8] Zeng Y., Priest C., Wang G., Wu G. (2020). Restoring the Nitrogen Cycle by Electrochemical Reduction of Nitrate: Progress and Prospects. Small Methods.

[9] Liu D., Qiao L., Peng S., Bai H., Liu C., Ip W.F., Lo K.H., Liu H., Ng K.W., Wang S. (2023). Recent Advances in Electrocatalysts for Efficient Nitrate Reduction to Ammonia. Adv. Funct. Mater..

[10] Liang X., Zhu H., Yang X., Xue S., Liang Z., Ren X., Liu A., Wu G. (2022). Recent Advances in Designing Efficient Electrocatalysts for Electrochemical Nitrate Reduction to Ammonia. Small Struct..

[11] Song Z., Qin L., Liu Y., Zhong Y., Guo Q., Geng Z., Zeng J. (2023). Efficient Electroreduction of Nitrate to Ammonia with CuPd Nanoalloy Catalysts. ChemSusChem.

[12] Qing G., Ghazfar R., Jackowski S.T., Habibzadeh F., Ashtiani M.M., Chen C.P., Smith M.R., Hamann T.W. (2020). Recent Advances and Challenges of Electrocatalytic N_2_ Reduction to Ammonia. Chem. Rev..

[13] Peng X., Mi Y., Bao H., Liu Y., Qi D., Qiu Y., Zhuo L., Zhao S., Sun J., Tang X. (2020). Ambient electrosynthesis of ammonia with efficient denitration. Nano Energy.

[14] Miao R., Chen D., Guo Z., Zhou Y., Chen C., Wang S. (2024). Recent advances in electrocatalytic upgrading of nitric oxide and beyond. Appl. Catal. B-Environ..

[15] Wu Z.Y., Karamad M., Yong X., Huang Q., Cullen D.A., Zhu P., Xia C., Xiao Q., Shakouri M., Chen F.Y. (2021). Electrochemical ammonia synthesis via nitrate reduction on Fe single atom catalyst. Nat. Commun..

[16] Chen D., Yin D., Zhang S., Yip S., Ho J.C. (2024). Nitrate electroreduction: Recent development in mechanistic understanding and electrocatalyst design. Mater. Today Energy.

[17] Jiang H., Chen G.F., Savateev O., Xue J., Ding L.X., Liang Z., Antonietti M., Wang H. (2023). Enabled Efficient Ammonia Synthesis and Energy Supply in a Zinc-Nitrate Battery System by Separating Nitrate Reduction Process into Two Stages. Angew. Chem. Int. Ed..

[18] Liu H., Xiang K., Yang B., Xie X., Wang D., Zhang C., Liu Z., Yang S., Liu C., Zou J. (2017). The electrochemical selective reduction of NO using CoSe_2_@CNTs hybrid. Environ. Sci. Pollut. Res. Int..

[19] Wang J., Zhao H., Haller G., Li Y. (2017). Recent advances in the selective catalytic reduction of NO*x* with NH_3_ on Cu-Chabazite catalysts. Appl. Catal. B Environ..

[20] Tursun M., Wu C. (2021). Vacancy-triggered and dopant-assisted NO electrocatalytic reduction over MoS_2_. Phys. Chem. Chem. Phys..

[21] Katsounaros I., Figueiredo M.C., Chen X., Calle-Vallejo F., Koper M.T.M. (2017). Structure- and Coverage-Sensitive Mechanism of NO Reduction on Platinum Electrodes. ACS Catal..

[22] Shi J., Wang C., Yang R., Chen F., Meng N., Yu Y., Zhang B. (2021). Promoting nitric oxide electroreduction to ammonia over electron-rich Cu modulated by Ru doping. Sci. China Chem..

[23] Tursun M., Wu C. (2021). NO Electroreduction by Transition Metal Dichalcogenides with Chalcogen Vacancies. ChemElectroChem.

[24] Ko B.H., Hasa B., Shin H., Zhao Y., Jiao F. (2022). Electrochemical Reduction of Gaseous Nitrogen Oxides on Transition Metals at Ambient Conditions. J. Am. Chem. Soc..

[25] Tursun M., Wu C. (2023). Defective 1T’-MoX_2_ (X = S, Se, Te) monolayers for electrocatalytic ammonia synthesis: Steric and electronic effects on the catalytic activity. Fuel.

[26] Clayborne A., Chun H.-J., Rankin R.B., Greeley J. (2015). Elucidation of Pathways for NO Electroreduction on Pt(111) from First Principles. Angew. Chem. Int. Ed..

[27] Cheng Q., Jiang Y.-X., Tian N., Zhou Z.-Y., Sun S.-G. (2010). Electrocatalytic reduction of nitric oxide on Pt nanocrystals of different shape in sulfuric acid solutions. Electrochim. Acta.

[28] Wang Z., Zhao J., Wang J., Cabrera C.R., Chen Z. (2018). A Co–N_4_ moiety embedded into graphene as an efficient single-atom-catalyst for NO electrochemical reduction: A computational study. J. Mater. Chem. A.

[29] Li H., Wu D., Wu J., Lv W., Duan Z., Ma D. (2024). Graphene-based iron single-atom catalysts for electrocatalytic nitric oxide reduction: A first-principles study. Nanoscale.

[30] Wang J., Li K., Hao Q., Liu D., Zhang X. (2023). Electroreduction NO to NH_3_ over single metal atom anchored on pyrrole type defective graphene: A DFT study. Chin. Chem. Lett..

[31] Wu Q., Huang B., Dai Y., Heine T., Ma Y. (2022). Main-group metal elements as promising active centers for single-atom catalyst toward nitric oxide reduction reaction. Npj 2d Mater. Appl..

[32] Tursun M., Wu C. (2022). Single Transition Metal Atoms Anchored on Defective MoS_2_ Monolayers for the Electrocatalytic Reduction of Nitric Oxide into Ammonia and Hydroxylamine. Inorg. Chem..

[33] Ruan W., Yang C., Hu J., Lin W., Guo X., Ding K. (2024). Investigation of a Single Atom Iron Catalyst for the Electrocatalytic Reduction of Nitric Oxide to Hydroxylamine: A DFT Study. Langmuir.

[34] Sun X., Zheng J., Yao Z., Deng S., Pan Z., Wang S., Wang J. (2022). DFT Investigation of Single Metal Atom-Doped 2D MA_2_Z_4_ Materials for NO Electrocatalytic Reduction to NH_3_. J. Phys. Chem. C.

[35] Xiao X., Cao Y., Hu L. (2025). First-principles study on single-layer electronic structure of Fe-doped MoS_2_ and the reduction of NO on the doped surface. Comput. Theor. Chem..

[36] Wu Y.-W., Wang H.-W., Wu Z.-L., Zhang X., Dong Y., Hu Z., Lv Y., Zhou X.-Y., Zhao L., Zhang B. (2025). Mechanism of NO electrocatalytic reduction over the MoS_2_-based single atom catalyst: A DFT investigation. Sep. Purif. Technol..

[37] Lin L., Pang D., Shi P., Xie K., Su L., Zhang Z. (2022). First-principles study of TM supported SnSe_2_ monolayer as an efficient electrocatalyst for NOER. Mol. Catal..

[38] Chen K., Zhang N., Wang F., Kang J., Chu K. (2023). Main-group indium single-atom catalysts for electrocatalytic NO reduction to NH_3_. J. Mater. Chem. A.

[39] Venkateswara Rao Nulakani N., Surya Kumar Choutipalli V., Akbar Ali M. (2025). Efficient electrocatalytic reduction of nitric oxide (NO) to ammonia (NH_3_) on metal-free B_4_@g-C_3_N_4_ nanosheet. Appl. Surf. Sci..

[40] Guo W., Tang X., Liao H., Peng J., Lian X. (2025). Theoretical screening of single-metal atom deposited on 2D BC_3_N_2_ monolayers for NO electrocatalytic reduction to NH_3_. Appl. Surf. Sci..

[41] Xiao Y., Shen C., Zhang W.B. (2022). Screening and prediction of metal-doped α-borophene monolayer for nitric oxide elimination. Mater. Today Chem..

[42] Yang L., Fan J., Zhu W. (2025). Single atom decorated wavy antimony nitride for nitric oxide degradation: A first-principles and machine learning study. Fuel.

[43] Wang J., Sun C., Sheng L., Zhuo Z., Li S., Wang J., Wang W., Sun J., Yang J., Xu K. Unveiling the electrocatalytic potential of main-group metal-embedded BC_3_ monolayer for highly efficient NO reduction to NH_3_. Chin. Chem. Lett..

[44] Niu H., Zhang Z., Wang X., Wan X., Kuai C., Guo Y. (2021). A Feasible Strategy for Identifying Single-Atom Catalysts Toward Electrochemical NO-to-NH_3_ Conversion. Small.

[45] He C.-Z., Zhang Y.-X., Wang J., Fu L. (2022). Anchor single atom in h-BN assist NO synthesis NH_3_: A computational view. Rare Met..

[46] Sun P.F., Wang W.L., Zhao X., Dang J.S. (2020). Defective h-BN sheet embedded atomic metals as highly active and selective electrocatalysts for NH_3_ fabrication via NO reduction. Phys. Chem. Chem. Phys..

[47] Fan J., Yang L., Zhu W. (2025). Transition metal-anchored BN tubes as single-atom catalysts for NO reduction reaction: A study of DFT and deep learning. Fuel.

[48] Wu J., Yu Y.X. (2022). A theoretical descriptor for screening efficient NO reduction electrocatalysts from transition-metal atoms on N-doped BP monolayer. J. Colloid. Interface Sci..

[49] Zang Y., Wu Q., Wang S., Huang B., Dai Y., Ma Y. (2023). Activating dual atomic electrocatalysts for the nitric oxide reduction reaction through the P/S element. Mater. Horiz..

[50] Liu S., Xing G., Liu J.-Y. (2023). Computational screening of single-atom catalysts for direct electrochemical NH_3_ synthesis from NO on defective boron phosphide monolayer. Appl. Surf. Sci..

[51] Tong T., Linghu Y., Wu G., Wang C., Wu C. (2022). Nitric oxide electrochemical reduction reaction on transition metal-doped MoSi_2_N_4_ monolayers. Phys. Chem. Chem. Phys..

[52] Wang X., Wu J., Zhang Y., Sun Y., Ma K., Xie Y., Zheng W., Tian Z., Kang Z., Zhang Y. (2023). Vacancy Defects in 2D Transition Metal Dichalcogenide Electrocatalysts: From Aggregated to Atomic Configuration. Adv. Mater..

[53] Zheng J., Lebedev K., Wu S., Huang C., Ayvali T., Wu T.S., Li Y., Ho P.L., Soo Y.L., Kirkland A. (2021). High Loading of Transition Metal Single Atoms on Chalcogenide Catalysts. J. Am. Chem. Soc..

[54] Tursun M., Abdukayum A., Wu C., Wang C. (2024). Screening WS_2_−based single−atom catalysts for electrocatalytic nitrate reduction to ammonia. Int. J. Hydrogen Energy.

[55] Kresse G., Furthmuller J. (1996). Efficient iterative schemes for ab initio total-energy calculations using a plane-wave basis set. Phys. Rev. B.

[56] Perdew J.P., Burke K., Ernzerhof M. (1996). Generalized gradient approximation made simple. Phys. Rev. Lett..

[57] Blochl P.E. (1994). Projector augmented-wave method. Phys. Rev. B.

[58] Grimme S., Antony J., Ehrlich S., Krieg H. (2010). A consistent and accurate ab initio parametrization of density functional dispersion correction (DFT-D) for the 94 elements H-Pu. J. Chem. Phys..

[59] Norskov J.K., Rossmeisl J., Logadottir A., Lindqvist L., Kitchin J.R., Bligaard T., Jónsson H. (2004). Origin of the overpotential for oxygen reduction at a fuel-cell cathode. J. Phys. Chem. B.

[60] Farberow C.A., Dumesic J.A., Mavrikakis M. (2014). Density Functional Theory Calculations and Analysis of Reaction Pathways for Reduction of Nitric Oxide by Hydrogen on Pt(111). ACS Catal..

[61] Wang V., Xu N., Liu J.-C., Tang G., Geng W.-T. (2021). VASPKIT: A user-friendly interface facilitating high-throughput computing and analysis using VASP code. Comput. Phys. Commun..

[62] Mathew K., Sundararaman R., Letchworth-Weaver K., Arias T.A., Hennig R.G. (2014). Implicit solvation model for density-functional study of nanocrystal surfaces and reaction pathways. J. Chem. Phys..

[63] Chen M., Zhu Z., Chen J., Xia L., Gan L., Zhou Y. (2024). Evaluating the efficiency of single-double atom catalysts in electrochemical NH_3_ production from NO based on CN monolayers. J. Mater. Chem. A.

[64] Zhu S., Zhang Y., Liu W., Yang D., Zhou G., Yang Z. (2025). Exploring the Catalytic Performance of Oxygen-Coordinated Single-Atom Catalysts for Nitric Oxide Electroreduction. J. Phys. Chem. C.

[65] Guo X., Lin S., Gu J., Zhang S., Chen Z., Huang S. (2020). Establishing a Theoretical Landscape for Identifying Basal Plane Active 2D Metal Borides (MBenes) toward Nitrogen Electroreduction. Adv. Funct. Mater..

[66] Wu Q., Wei W., Lv X., Wang Y., Huang B., Dai Y. (2019). Cu@g-C_3_N_4_: An Efficient Single-Atom Electrocatalyst for NO Electrochemical Reduction with Suppressed Hydrogen Evolution. J. Phys. Chem. C.

[67] Lin L., Yan L., Fu L., He C., Xie K., Zhu L., Sun J., Zhang Z. (2022). First principle investigation of W/P3C sheet as an efficient single atom electrocatalyst for N_2_ and NO electrochemical reaction with suppressed hydrogen evolution. Fuel.

[68] Liu L., Zuo Z.J., Du Y., Wu T., Wu J., Gao J., Mu T., Zhang Y.C., Zhu X.D. (2025). Role of synergies of Cu/Fe_3_O_4_ electrocatalyst for nitric oxide reduction to ammonia. J. Colloid Interface Sci..

