# Supplementary material for: Boosting the Performance of Electrocatalytic NO Reduction to NH3 by Decorating WS2 with Single Transition Metal Atoms: A DFT Study
Source: Materials (Basel). 2025 May 17;18(10):2341. doi: 10.3390/ma18102341 (PMC12113338; doi:10.3390/ma18102341)
Supplement: Supplementary file 1 [file materials-18-02341-s001.zip › materials-3570588-supplementary.pdf]

Supporting Information

Boosting the performance of electrocatalytic NO reduction to NH_3 by decorating WS_2 with single transition metal atoms: A DFT study

Mamutjan Tursun [1,], Ayxamgul Abduryim [1], and Chao Wu [2,]**

[1] Xinjiang Key Laboratory of Novel Functional Materials Chemistry, College of chemistry and Environmental Sciences, Kashi University, Kashi 844000, China.

[2] Frontier Institute of Science and Technology, Xi'an Jiaotong University, Xi'an 710054, China.

Contents

Figure S1 AIMD simulations for Zn@WS_2 1

Figure S2 Free energy diagrams of eNORR over defective WS_2 catalyst 2

Table S1 The adsorption energy of NO over TM@WS_2 catalysts with different patterns 3

Table S2 Limiting potentials of eNORR toward NH_3 over different single atom catalysts. 4

References 5

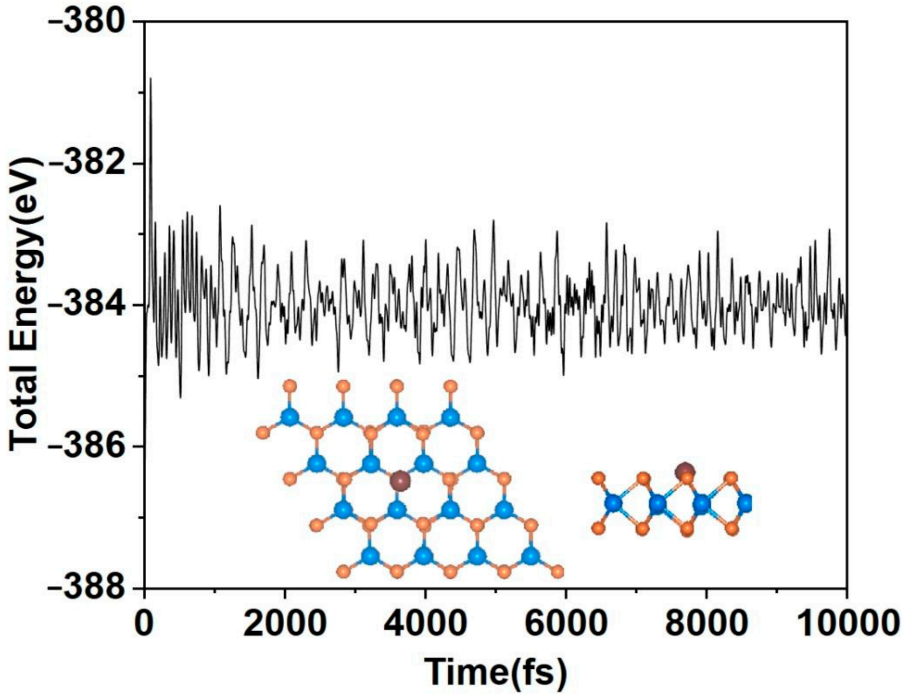

Figure S1. Ab initio molecular dynamics (AIMD) simulations at 500 K forZn@WS_2 catalysts.

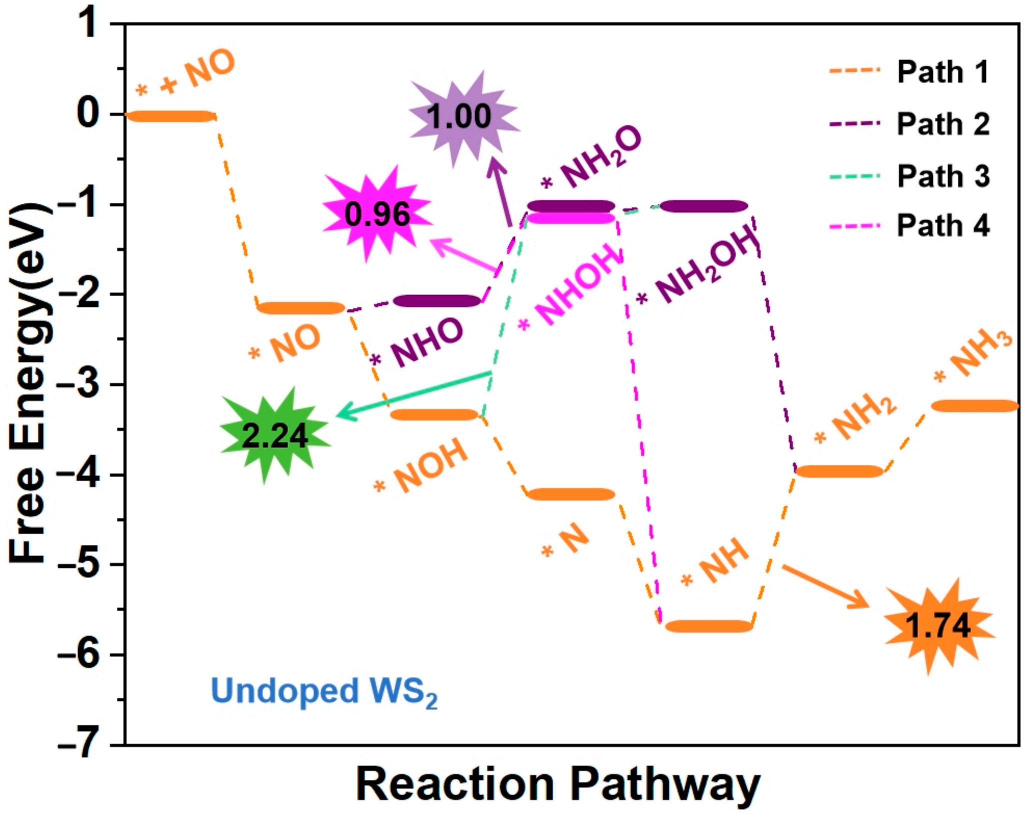

Figure S2. Free energy diagrams of eNORR over undoped defective WS_2 catalyst.

Table S1 The Gibbs energies of NO adsorptipon on $TM@WS_2$ catalysts via different configurations (eV).

TM	N-end	NO-side	O-end
Sc	-1.64	-1.32	-0.96
Ti	-1.57	-1.25	-0.80
Mn	-1.42	-1.09	-0.61
Fe	-2.44	-1.81	-1.16
Co	-2.50	//	-0.94
Ni	-1.20	//	-0.22
Cu	-1.26	//	-0.54
Zn	-1.26	//	-0.44
Y	-1.39	-0.96	-0.79
Zr	-2.03	-1.82	-1.11
Rh	-2.17	//	-0.52
Pd	-1.23	//	-0.55
Ag	-0.74	//	-0.25
Hf	-1.50	-1.30	-0.94
Pt	-0.81	//	0.11
Au	-0.98	//	-0.27

// denotes that the adsorption configuration is unstable. It transforms into the N-end configuration.

Table S2 Limiting potentials of eNORR toward NH_3 over different single atom catalysts.

Catalysts	Calculated U_L (V)	limiting steps	References
Ti@WS_2	0	//	This work
Zr@WS_2	0	//	This work
Hf@WS_2	-0.15	NO→ NOH	This work
Mn@WS_2	-0.19	NO → NHO	This work
Co@WS_2	-0.26	NO → NHO	This work
Co–N_4/graphene	-0.12	NO → NHO	[1]
Zr-C_2N	-0.33	NO→ NOH	[2]
Cu@g-C_3N_4	-0.37	NO → NHO	[3]
W/P_3C	-0. 59	NH_2→ NH_3	[4]
Cu@hexagonal boron nitride	-0.23	NO → NHO	[5]
Cu/Fe_3O_4	-0.5	NO→NOH	[6]
Zr@$SnSe_2$	-0.11	NO→NHO	[7]
Fe–N_4/graphene	0	//	[8]
Pd@MoS_2	-0.38	NO→NHO	[9]
Gr/$HfSi_2N_4$	-0.11	NH_2→NH_3	[10]
In/a-MoO_3	-0.20	NO→NHO	[11]
Rh@BC_3N_2	-0.37	NO→NHO	[12]
Nb@SbN	-0.06	NO→NHO	[13]
Pd@@B_2N_2	-0.55	NH_2→ NH_3	[14]
Mn@BN	-0.23	NH_2→ NH_3	[15]
MoN_3/BP	-0.10	NO→ NOH	[16]
Pt@$MoSi_2N_4$	-0.10	NH_2→ NH_3	[17]

Notes: // denotes that there is no potential limiting step in the favourable reaction pathway.

References

1. Wang, Z.; Zhao, J.; Wang, J.; Cabrera, C. R.; Chen, Z., A Co–N_4 moiety embedded into graphene as an efficient single-atom-catalyst for NO electrochemical reduction: a computational study. J Mater Chem A 2018, 6, (17), 7547-7556.
2. Niu, H.; Zhang, Z.; Wang, X.; Wan, X.; Kuai, C.; Guo, Y., A Feasible Strategy for Identifying Single-Atom Catalysts Toward Electrochemical NO-to-NH_3 Conversion. Small 2021, 17, (36), e2102396.
3. Wu, Q.; Wei, W.; Lv, X.; Wang, Y.; Huang, B.; Dai, Y., Cu@g-C_3N_4: An Efficient Single-Atom Electrocatalyst for NO Electrochemical Reduction with Suppressed Hydrogen Evolution. J Phys Chem C 2019, 123, (51), 31043-31049.
4. Lin, L.; Yan, L.; Fu, L.; He, C.; Xie, K.; Zhu, L.; Sun, J.; Zhang, Z., First principle investigation of W/P3C sheet as an efficient single atom electrocatalyst for N_2 and NO electrochemical reaction with suppressed hydrogen evolution. Fuel 2022, 308.
5. Sun, P. F.; Wang, W. L.; Zhao, X.; Dang, J. S., Defective h-BN sheet embedded atomic metals as highly active and selective electrocatalysts for NH_3 fabrication via NO reduction. Phys Chem Chem Phys 2020, 22, (39), 22627-22634.
6. Liu, L.; Zuo, Z. J.; Du, Y.; Wu, T.; Wu, J.; Gao, J.; Mu, T.; Zhang, Y. C.; Zhu, X. D., Role of synergies of Cu/Fe3O4 electrocatalyst for nitric oxide reduction to ammonia. J Colloid Interface Sci 2025, 691, 137376.
7. Lin, L.; Pang, D.; Shi, P.; Xie, K.; Su, L.; Zhang, Z., First-principles study of TM supported $SnSe_2$ monolayer as an efficient electrocatalyst for NOER. Mol Catal 2022, 533.
8. Wang, J.; Li, K.; Hao, Q.; Liu, D.; Zhang, X., Electroreduction NO to NH_3 over single metal atom anchored on pyrrole type defective graphene: A DFT study. Chinese Chem Lett 2023, 34, (5).
9. Tursun, M.; Wu, C., Single Transition Metal Atoms Anchored on Defective MoS2 Monolayers for the Electrocatalytic Reduction of Nitric Oxide into Ammonia and Hydroxylamine. Inorg Chem 2022, 61, (44), 17448-17458.
10. Sun, X.; Zheng, J.; Yao, Z.; Deng, S.; Pan, Z.; Wang, S.; Wang, J., DFT Investigation of Single Metal Atom-Doped 2D MA_2Z_4 Materials for NO Electrocatalytic Reduction to NH_3. J Phys Chem C 2022, 126, (41), 17598-17607.
11. Chen, K.; Zhang, N.; Wang, F.; Kang, J.; Chu, K., Main-group indium single-atom catalysts for electrocatalytic NO reduction to NH_3. J Mater Chem A 2023, 11, (13), 6814-6819.
12. Guo, W.; Tang, X.; Liao, H.; Peng, J.; Lian, X., Theoretical screening of single-metal atom deposited on 2D BC_3N_2 monolayers for NO electrocatalytic reduction to NH_3. Appl Surf Sci 2025, 690.
13. Yang, L.; Fan, J.; Zhu, W., Single atom decorated wavy antimony nitride for nitric oxide degradation: A first-principles and machine learning study. Fuel 2025, 380.
14. He, C.-Z.; Zhang, Y.-X.; Wang, J.; Fu, L., Anchor single atom in h-BN assist NO synthesis NH_3: a computational view. Rare Metals 2022, 41, (10), 3456-3465.
15. Fan, J.; Yang, L.; Zhu, W., Transition metal-anchored BN tubes as single-atom catalysts for NO reduction reaction: A study of DFT and deep learning. Fuel 2025,

386.
16. Wu, J.; Yu, Y. X., A theoretical descriptor for screening efficient NO reduction electrocatalysts from transition-metal atoms on N-doped BP monolayer. J Colloid Interface Sci 2022, 623, 432-444.
17. Tong, T.; Linghu, Y.; Wu, G.; Wang, C.; Wu, C., Nitric oxide electrochemical reduction reaction on transition metal-doped $MoSi_2N_4$ monolayers. Phys Chem Chem Phys 2022, 24, (31), 18943-18951.
